# Elongation at Midcell in Preparation of Cell Division Requires FtsZ, but Not MreB nor PBP2 in *Caulobacter crescentus*

**DOI:** 10.3389/fmicb.2021.732031

**Published:** 2021-08-27

**Authors:** Muriel C. F. van Teeseling

**Affiliations:** ^1^Junior Research Group Prokaryotic Cell Biology, Department Microbial Interactions, Institute of Microbiology, Friedrich-Schiller-Universität, Jena, Germany; ^2^Department of Biology, University of Marburg, Marburg, Germany

**Keywords:** medial elongation, PBP3-independent peptidoglycan synthesis (PIPS), preseptal peptidoglycan synthesis, cell wall, peptidoglycan, elongasome, divisome

## Abstract

Controlled growth of the cell wall is a key prerequisite for bacterial cell division. The existing view of the canonical rod-shaped bacterial cell dictates that newborn cells first elongate throughout their side walls using the elongasome protein complex, and subsequently use the divisome to coordinate constriction of the dividing daughter cells. Interestingly, another growth phase has been observed in between elongasome-mediated elongation and constriction, during which the cell elongates from the midcell outward. This growth phase, that has been observed in *E*scherichia *coli* and *Caulobacter crescentus*, remains severely understudied and its mechanisms remain elusive. One pressing open question is which role the elongasome key-component MreB plays in this respect. This study quantitatively investigates this growth phase in *C. crescentus* and focuses on the role of both divisome and elongasome components. This growth phase is found to initiate well after MreB localizes at midcell, although it does not require its presence at this subcellular location nor the action of key elongasome components. Instead, the divisome component FtsZ seems to be required for elongation at midcell. This study thus shines more light on this growth phase in an important model organism and paves the road to more in-depth studies.

## Introduction

Almost all bacterial cells rely on the cell-spanning macromolecule peptidoglycan (PG) to provide integrity and shape their cells (de Pedro and Cava, 2015; van Teeseling et al., 2017). It is therefore of key importance for bacteria to tightly control growth and remodeling of its PG sacculus throughout their cell cycle (Typas et al., 2012; Egan et al., 2020). The cell cycle of most bacterial cells involves multiple growth modes, and generally involves that the cells first elongate before they start the process of constriction that effectuates cell division (Randich and Brun, 2015; Kysela et al., 2016). Although some bacteria elongate from one or both poles [e.g., some *Alphaproteobacteria* (Brown et al., 2012) and *Actinobacteria* (Umeda and Amako, 1983; Flärdh, 2010)], most bacteria grow longer by adding new cell wall dispersed throughout their side walls (Kysela et al., 2016). This dispersed elongation mechanism has been studied in great detail, which unveiled an underlying multi-protein complex called the elongasome. The elongasome harbors the scaffolding protein MreB, proteins involved in PG synthesis (e.g., RodA and PBP2), as well as proteins that break existing PG bonds to make space for insertion of new material (Egan et al., 2020). It is clear that MreB is a crucial part of the elongasome, but its exact role remains puzzling (Ducret and Grangeasse, 2021). Instead of a scaffold that actively directs the movement of all other elongasome proteins, MreB seems to have a subtler coordinating role (Dion et al., 2019; Dersch et al., 2020), in which its movement in part seems to depend on PG synthases that take the lead (Dominguez-Escobar et al., 2011; Garner et al., 2011; van Teeffelen et al., 2011; Özbaykal et al., 2020).

The other important growth phase taking place in bacteria is the constricting process, which incorporates cell wall material at midcell, thereby decreasing the diameter of the cell while generating the new poles. In parallel to the elongasome, this growth phase is supported by a dedicated multiprotein complex, called the divisome. In addition to specific PG synthase (e.g., FtsW and PBP3) and hydrolase enzymes, this complex features the scaffolding protein FtsZ (Egan et al., 2020). Very recent studies point toward two divisome subcomplexes, one consisting of the treadmilling FtsZ (Bisson Filho et al., 2017; Yang et al., 2017) with its anchors (Squyres et al., 2021) that seems to act as a resting stage for the second subcomplex. This second subcomplex consists of PG synthases and seems to be active when it moves around the circumference of the cell as a separate subcomplex (Yang et al., 2021). Interestingly, it seems that after constriction has passed a certain threshold with the help of FtsZ, constriction is finished by the synthase subcomplex (Monteiro et al., 2018; Silber et al., 2021).

Apart from these two thoroughly studied growth phases, some model organisms display an additional growth phase that takes place after elongasome-guided elongation and before divisome-guided constriction. During this process, that has been first described in *E*scherichia *coli* (Wientjes and Nanninga, 1989), the cells elongate exclusively from the midcell outward. Interestingly, this process has been scarcely studied. One observation that might start to explain what happens in the transition from elongasome to divisome-guided growth, is that, at least in *E. coli*, FtsZ arrives at midcell and is then flanked by rings of divisome components, including MreB (Vats and Rothfield, 2007; Vats et al., 2009). This led to the hypothesis that either MreB itself guides growth at midcell or it transfers other elongasome components to FtsZ that then steers elongation at midcell (Potluri et al., 2012). Interestingly, however, all of the tested divisome and elongasome proteins, except for FtsZ and its anchor ZipA were found dispensable for this growth mode in *E. coli* (de Pedro et al., 1997; Varma and Young, 2009; Potluri et al., 2012). Most notably, *E. coli* was shown to still grow *via* medial elongation when the divisome-specific essential PG synthase PBP3, and MreB and its cognate synthase PBP2 were absent or non-functional. More in-depth studies in *E. coli* suggest that a very early divisome consisting of FtsZ anchored by ZipA and FtsA recruits the PG synthases PBP1A and 1B to midcell to drive this preseptal elongation [which has also been described as PBP3-independent peptidoglycan synthesis (PIPS)] (Pazos et al., 2018). It remains puzzling, however, if there is any role for MreB, which after all forms rings next to, and even directly interacts with, FtsZ (Fenton and Gerdes, 2013), shortly before preseptal PG synthesis commences.

Although it remains unclear how widespread this growth phase is, a similar growth phase has been observed in another Gram-negative model organism: *Caulobacter crescentus* (Aaron et al., 2007). Interestingly, in this species a relatively large part of the elongation takes place by the medial elongation growth phase (as it is called in *C. crescentus*). As in *E. coli*, medial elongation in *C. crescentus* requires FtsZ. It furthermore seems that PG synthesis at midcell starts shortly after the arrival of FtsZ at midcell. Also in *C. crescentus*, MreB was seen to form a band at midcell, dependent on FtsZs localization at this site (Figge et al., 2004; Gitai et al., 2004). Indirect evidence suggested that MreBs arrival at midcell is followed quickly by the onset of medial elongation (Aaron et al., 2007). A more in-depth study is needed to further elucidate the role of MreB in medial elongation in *C. crescentus*, as it is believed to play a role in this process by some (Randich and Brun, 2015), but non-quantitative indications by others (Aaron et al., 2007) show it might not be necessary for medial elongation. Furthermore, it remains unclear which PG synthases are involved. All in all, the mechanisms of this growth phase remain puzzling and it is an open question if the growth phases in *E. coli* and *C. crescentus* are mechanistically the same process. In order to build a base toward better understanding of the importance and mechanisms of this growth phase, these questions should be addressed.

This study thus focuses on medial elongation in *C. crescentus* and attempts to build a quantitative base of information about the growth phase in this model organism. Specifically, the timing of events surrounding MreBs arrival at midcell and the onset of medial elongation are elucidated. Furthermore, the importance of MreB for this growth phase is studied and quantified using multiple approaches. Additionally, it is shown that the elongasome-specific PBP2 is not required for this mode of growth, whereas the presence of FtsZ is crucial. It thus seems that preseptal PG synthesis in *E. coli* and medial elongation in *C. crescentus* are governed by the same or very similar molecular mechanisms.

## Materials and Methods

### Bacterial Strains and Growth Conditions

All strains that are analyzed in this study are derivatives of the synchronizable *C. crescentus* wild-type CB15N (Evinger and Agabian, 1977; Marks et al., 2010). Strain MT309 carries a chromosomally integrated *venus-mreB* under the control of the *xylX* promoter (Billini et al., 2019). Strain JAT790 contains the *mreB* variant G165A at the native *mreB* locus and a fluorescently labeled copy of the same mreB variant *venus-_*G*165*A*_mreB* under the control of the *xylX* promoter (Dye et al., 2011). Another mreB variant, *mreB*_*Q*26*P*_, is present at the native locus instead of the normal MreB in strain CJW1715 (Aaron et al., 2007). In the FtsZ depletion strain YB1585, the sole full copy of *ftsZ* is chromosomally integrated under the control of the *xylX* promotor, whereas a non-functional truncated version (consisting of the first 163 codons) is present at the native locus of *ftsZ* (Wang et al., 2001). Strain information is detailed in Table 1.

**TABLE 1:** Description of all strains used in this study.

**Strain**	**Genotype**	**Source**
CB15N	*C. crescentus* wild-type	Evinger and Agabian, 1977; Marks et al., 2010
CJW1715	CB15N P_*mreB*_:*mreB*_*Q*26*P*_	Aaron et al., 2007
JAT790	CB15N P_*mreB*_:*mreB*_*G*165*A*_ P_*xyl*_:*venus-mreB*_*G*165*A*_	Dye et al., 2011
MT309	CB15N P*_*xyl*_*:*venus-mreB*	Billini et al., 2019
YB1585	CB15N P*_*ftsZ*_*:P*_*xyl*_*-*ftsZ*	Wang et al., 2001

All strains were cultivated in peptone-yeast extract (PYE) medium (Pointdexter, 1964) at 28°C, while shaking at 210 rpm. Gene expression from the xylose-inducible *xylX* promoter (Meisenzahl et al., 1997) was induced for 75 min with 0.03% xylose. The following inhibitors were used: A22 (10 μg/ml) and mecillinam (150 μg/ml).

Synchronization of *C. crescentus* was achieved by density gradient centrifugation, using Percoll (Tsai and Alley, 2001). After synchronization, cells were released in PYE medium (with 0.03% xylose and/or inhibitors, when applicable) and allowed to grow at 28°C, while shaking at 210 rpm.

### Peptidoglycan Labeling

To indicate areas of active PG insertion and remodeling, cells were stained with the fluorescent dye hydroxycoumarin-carbonyl-amino-D-alanine (HADA) (Kuru et al., 2012). For this, cells at multiple time points after synchronization were incubated with 500 μM HADA in PYE (including 0.03% xylose, where applicable) while shaking at 300 rpm for 2 min at 28°C. Cells were then washed once in PYE medium without HADA and immediately prepared for imaging.

### Microscopy and Image Analysis

After synchronization and PG labeling, cells were immobilized on pads consisting of 1% agar in water (to prevent further growth) and immediately imaged with an Axio Observer Z1 microscope (Zeiss, Germany). Samples were excited with the use of an X-Cite 120PC metal halide light source (EXFO, Canada) and ET-YFP and/or ET-DAPI filter cubes (Chroma, United States) were used for fluorescence detection. Images were obtained through a Zeiss Plan-Apochromat 100×/1.4 oil immersion Ph3 objective and recorded with a pco.edge sCMOS camera (PCO, Germany) using VisiView (Visitron Systems, Germany) software. Dimensions of the cells (length and width) and their fluorescent properties were analyzed with the image analysis software tool BacStalk (Hartmann et al., 2020) using standard settings for stalked cells. All cells were manually inspected to remove wrongly detected cells. The remaining cell outlines generated by BacStalk, as well as the underlying phase contrast images, were inspected by eye to determine if cells were constricting. For each replicate, a number of cells was analyzed until 100 non-constricting cells were present in the analysis, next to a varying number of constricting cells (between 0 and 80, dependent on the conditions). Each cell was then inspected to determine if HADA and/or fluorescently labeled MreB had accumulated at midcell (the midcell region was determined by eye) in respect to the background. The maxima of the different fluorescent channels per cell were extracted using BacStalk. In order to compare the percentage of cells undergoing medial elongation in the different conditions, the fraction of non-constricting cells (*n* = 100 per replicate) that shows HADA at midcell (either in the presence or absence) was evaluated, as well as the information if a fluorescently tagged MreB (in the relevant strains) was present at midcell in the same cell. Measurements were exported to Excel 2016 (Microsoft, United States), where average and standard deviations were calculated. Images were processed using Fiji (Schindelin et al., 2012) and Adobe Illustrator CS5 (Adobe Systems, United States). Graphs representing cellular dimensions were created using SuperPlotsOfData (Goedhart, 2021) and show constricting, as well as non-constricting cells.

## Results

### Onset of Medial Elongation Takes Place After MreB Accumulation at Midcell

**FIGURE 1:**
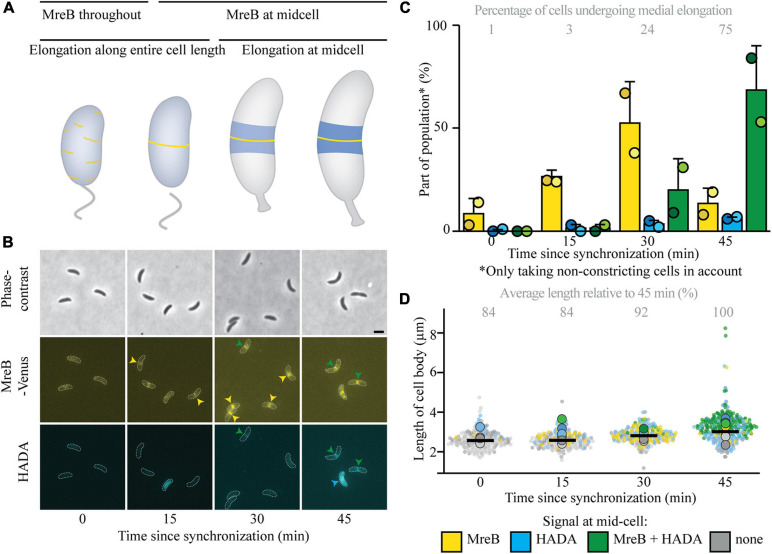
MreB accumulates at midcell clearly before elongation at midcell commences. Intracellular localization of MreB (yellow) and peptidoglycan insertion and remodeling (blue) summarized in a cartoon **(A)**, based on the experiments shown in **(B–D)**. Light microscopy images **(B)** and respective quantification **(C)** of strain MT309 (P_xyl_-*venus-mreB*) grown for the indicated duration after synchronization, stained with a short pulse of HADA show that with time MreB accumulates at midcell and cell wall remodeling at midcell commences with a delay. Cells in the representative images **(B)** are outlined and midcell localization is indicated by arrowheads: colocalizing MreB and HADA are indicated in green, only MreB in yellow, and only HADA in blue. Quantification of fluorescence signals **(C)** at midcell (using the color scheme outlined above) was performed on 100 non-constricting cells per replicate for two replicates per timepoint (value for each replicate is indicated by circles, replicate 1 is shown in a darker shade and replicate 2 in a light shade). Bars indicate the average of two replicates and error bars indicate standard deviation. In addition, the average percentage of non-constricting cells showing HADA (either in presence or absence of MreB) is given for each timepoint. Analysis of the length of cell bodies (of both constricting and non-constricting cells, in total at least 100 cells per replicate) shows that cells become longer with time and cells that are incorporating HADA are relatively longer **(D)**. Cell lengths are shown in superplots, where each small dot indicates the value for a single cell. The color of each dot follows the color scheme outlined above, in order to identify differences in cell length for the different subpopulations, and shows different shades to differentiate between the replicates. The large dots show the average cell length for each localization subpopulation per replicate. The horizontal black bar indicates the average length over all cells and the numbers above the graph express this average length as a percentage of the average length of the cells after 45 min.

One of the biggest outstanding questions concerning medial elongation is if it depends on the presence of the elongasome component MreB at midcell. As a first step in addressing this question, the relative timing of arrival of MreB at midcell versus the onset of medial elongation was analyzed. For this, *C. crescentus* cells expressing a fluorescent fusion to MreB were synchronized and stained with a short pulse of the fluorescent PG precursor HADA to visualize both the intracellular location of MreB and PG insertion in the same cells. This analysis showed that in almost all cells, MreB arrives at midcell before medial elongation commences (Figure 1 and Supplementary Figure 1). MreB starts to accumulate at midcell already quite early in the cell cycle, as 9 and 28% of cells respectively 0 and 15 min after synchronization show MreB at midcell (Figures 1B,C). Elongation at midcell starts later, as 3 and 24% of the cells show a HADA enrichment at midcell respectively 15 min and 30 min after synchronization. With time and as cells get longer, the fraction of (non-constricting) cells showing HADA incorporation at midcell, as well as the maximum intensity of the HADA signal increases (Supplementary Figure 1A), indicating that medial elongation takes over as a growth phase (Figures 1C,D). The sequence of events therefore seems to be that cells first recruit MreB to midcell, and as they become longer PG insertion then starts at midcell, where MreB is still present, as most cells that show HADA signal also have MreB at midcell, at least in the early stages. As HADA signal at midcell is very scarcely seen without MreB being at midcell at early stages, it remains unclear if the presence of elongasome components at midcell is a prerequisite for medial elongation or if medial elongation takes place independently.

### Medial Elongation Can Start but Proceeds Slower (at the Population Level) When MreB’s Arrival to Midcell Is Delayed

**FIGURE 2:**
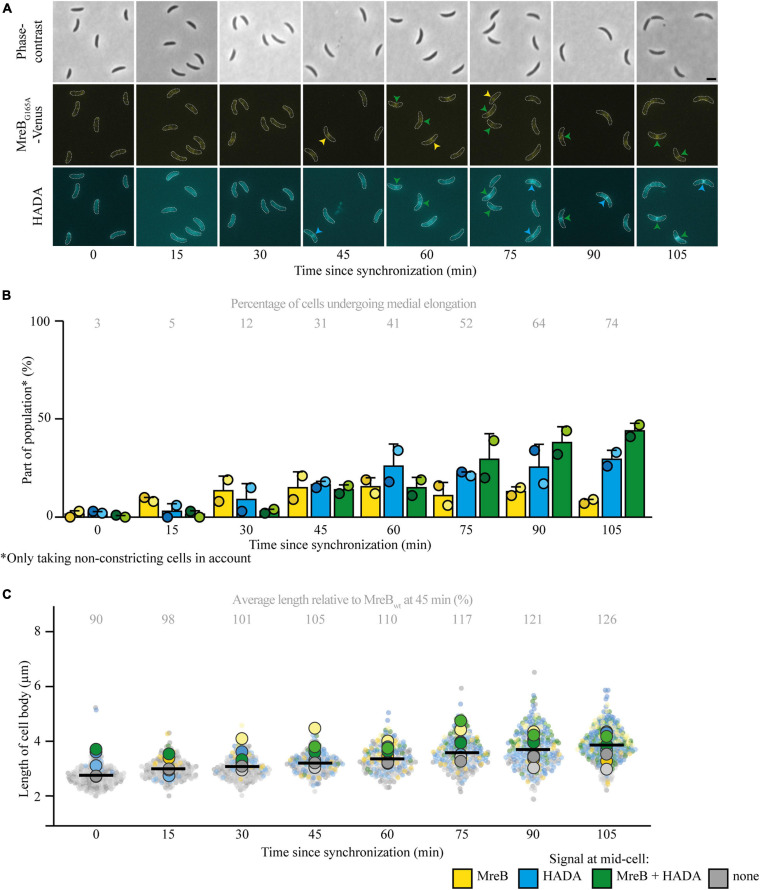
Medial elongation can precede MreB accumulation at midcell in a strain with delayed MreB recruitment. Light microscopy images **(A)** and respective quantification **(B)** of strain JAT790 (P_mreB_-*_G165A_mreB* P_xyl_-*venus-_G165A_mreB*) grown for the indicated duration after synchronization, stained with a short pulse of HADA show that medial elongation commences in the absence of MreB at midcell in part of the population. Cells in the representative images **(A)** are outlined and midcell localization is indicated by arrowheads: colocalizing MreB and HADA are indicated in green, only MreB in yellow, and only HADA in blue. Quantification of fluorescence signals **(B)** at midcell (using the color scheme outlined above) was performed on 100 non-constricting cells per replicate for two replicates per timepoint (value for each replicate is indicated by circles, replicate 1 is shown in a darker shade and replicate 2 in a light shade). Bars indicate the average of two replicates and error bars indicate standard deviation. In addition, the average percentage of non-constricting cells showing HADA (either in presence or absence of MreB) is given for each timepoint. Analysis of the length of cell bodies (of both constricting and non-constricting cells, in total at least 100 cells per replicate) shows that cells become longer with time and cells that are incorporating HADA are relatively longer **(C)**. Cell lengths are shown in superplots, where each small dot indicates the value for a single cell. The color of each dot follows the color scheme outlined above, in order to identify differences in cell length for the different subpopulations, and shows different shades to differentiate between the replicates. The large dots show the average cell length for each localization subpopulation per replicate. The horizontal black bar indicates the average length over all cells and the numbers above the graph express this average length as a percentage of the average length of the cells carrying MreB_*wt*_ after 45 min (strain MT309, indicated in Figure 1).

To answer the question if medial elongation can only take place when elongasome components, specifically MreB, are present at midcell, additional experiments were performed. As a first step, a strain expressing the MreB variant MreB^G165A^ instead of the native MreB (and an inducible MreB^G165A^ fused to a fluorescent protein) was investigated (Figure 2 and Supplementary Figure 2), as this strain was previously shown to have a delayed recruitment of MreB to midcell and a longer cell cycle (Dye et al., 2011). Indeed, it could be confirmed that MreB arrived later at midcell by approximately 15 min, as respectively 11 and 17% of cells showed MreB localization at 15 and 30 min (Figures 2A,B). Interestingly, the onset of medial elongation was comparable to a strain with native MreB, with respectively 5 and 12% of cells showing HADA at midcell 15 and 30 min after synchronization (Figure 2B). Like in the strain with the native MreB variant, with time and increasing cell length (Figure 2C), more and more (non-constricting) elongating cells show a HADA accumulation, with a higher maximum intensity, at midcell. However, the fraction of cells that undergoes medial elongation and the maximum HADA intensity increase slower in the strain with the delayed MreB variant than in the wild-type MreB (compare Figure 1C with Figure 2B and Supplementary Figure 1A with Supplementary Figure 2A). This population-wide delay of medial elongation becomes very clear when comparing the timepoint at which 75% of non-constricting cells shows a HADA signal at midcell: for cells expressing MreB_wt_ this takes place 45 min after synchronization and for cells with MreB^G165A^ this percentage is reached only slightly before 105 min after synchronization, at which point the cells had more time to elongate and were on average longer than their counterparts expressing MreB_wt_. Taken together, the observations that (a) HADA accumulation at midcell can take place before MreB reaches the same location and (b) a considerable fraction of cells shows HADA signal at midcell in the absence of MreB (Figure 2B) suggests that medial elongation does not require the presence of MreB at midcell, although a timely presence of MreB does speed up medial elongation at the population level.

### Medial Elongation Takes Place in the Absence of MreB and PBP2 at Midcell

**FIGURE 3:**
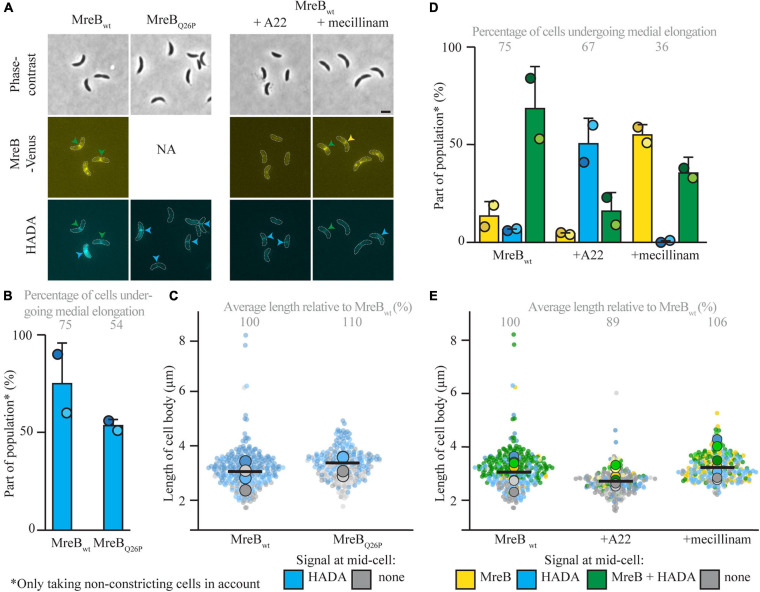
Medial elongation can take place in the absence of elongasome components at midcell. Light microscopy images **(A)** and respective quantification of strains CJW1715 (P_mreB_-*_Q26P_mreB*) **(B)** and MT309 (P_xyl_-*venus-mreB*), synchronized and subsequently grown in the absence of additives and (for MT309) in the presence of the MreB inhibitor A22 (10 μg/ml) or the PBP2 inhibitor mecillinam (150 μg/ml) **(D)**. Cells were harvested 45 min after synchronization and subjected to a short pulse HADA staining. Cells in the representative images **(A)** are outlined and midcell localization is indicated by arrowheads: colocalizing MreB and HADA are indicated in green, only MreB in yellow and only HADA in blue. Quantification of fluorescence signals **(B,D)** at midcell (using the color scheme outlined above, with the exception that in the case of strain CJW1715 MreB was not fluorescently labeled, and therefore only blue can be used) was performed on 100 non-constricting cells per replicate for two replicates per timepoint (value for each replicate is indicated by circles, replicate 1 is shown in a darker shade and replicate 2 in a light shade). Bars indicate the average of two replicates and error bars indicate standard deviation. In addition, the average percentage of non-constricting cells showing HADA (either in presence or absence of MreB) is given for each timepoint. Data shown for MreB_wt_ in both cases are from the same two replicates of strain MT309 (also shown in Figure 1). Analysis of the length of the cell bodies (of both constricting and non-constricting cells, in total at least 100 cells per replicate) shows that cells expressing MreB_Q26P_ are slightly longer than cells expressing MreB_wt_ **(C)**. Analysis of the length of the cell bodies (of both constricting and non-constricting cells, in total at least 100 cells per replicate) for cells of strain MT309 incubated without additives, shows that with A22 cells are a bit shorter and with mecillinam cells are slightly longer **(E)**. Cell lengths are shown in superplots, where each small dot indicates the value for a single cell. The color of each dot follows the color scheme outlined above, in order to identify differences in cell length for the different subpopulations, and shows different shades to differentiate between the replicates. The large dots show the average cell length for each localization subpopulation per replicate. The horizontal black bar indicates the average length over all cells and the numbers above the graph express this average length as a percentage of the average length of the cells carrying MreB_wt_ after 45 min (strain MT309, indicated in Figure 1).

To further look into the role of MreB and other elongasome components for the onset of medial elongation, multiple parallel routes were taken. First, a strain carrying a MreB variant (MreB_Q26P_) that does not get recruited to midcell at all (Aaron et al., 2007) was followed. This experiment confirmed that medial elongation can take place without MreB being present at midcell, as the majority of cells showed a HADA signal at midcell 45 min after synchronization (Figures 3A,B). As compared to cells expressing MreB_wt_ the cell length of this strain was slightly longer, again showing that MreB localization at midcell is not needed for cellular elongation (Figure 3C). The fraction of cells that shows medial elongation [and the maximum intensity of the HADA signal (Supplementary Figure 3A)] is lower than in strain MT309 at the same timepoint, this might be caused by a delay in the cell cycle, indicated by a lower percentage of constricting cells (Supplementary Figure 3C). In parallel, the localization of MreB_wt_ and HADA where studied upon treatment of strain MT309 (P_xyl_-*venus-mreB*) with the MreB inhibitor A22 (Figures 3A,D,E and Supplementary Figures 3B,D,E). As expected, only a low fraction of cells showed MreB localization after treatment with A22 (Figure 3D) and cells became slightly wider (Supplementary Figure 3E) as is typical for cells treated with A22 (Tropini et al., 2014). In line with the previous results on MreB variants, also upon A22 treatment cells showed medial elongation (as shown by the accumulation of HADA, either in the absence or presence of MreB, at midcell), almost up to the same extent as cells untreated with A22 (Figure 3D).

The elongasome does not only consist of the actin-homolog MreB, but includes multiple proteins involved in PG synthesis and remodeling, such as the elongasome-specific synthase PBP2. PBP2 was shown to colocalize with FtsZ and MreB at midcell in *C. crescentus* cells that have undergone an osmotic challenge (Hocking et al., 2012). This localization was shown to be dependent on FtsZ, but independent on MreB, which urges the question if PBP2 might be involved in medial elongation. To investigate this question, PG incorporation and MreB_wt_ localization were followed in the presence of the PBP2 inhibitor mecillinam (Figures 3A,D,E and Supplementary Figures 3B,D,E). Upon treatment with mecillinam, the cell width increased as compared to untreated cells, as has been reported before for mecillinam treatment (Tropini et al., 2014). The results were similar to these for MreB: medial elongation still took place upon inhibition of PBP2, suggesting that medial elongation does not depend on this PBP. The fraction of cells that showed medial elongation was lower as compared to samples without mecillinam, but this might well be caused by a short delay in the cell cycle [also exemplified by a drop in the percentage of constricting cells as compared to the untreated strain (Supplementary Figure 3D)] as mecillinam inhibits PBP2 that normally aids in dispersed elongation. All in all, it becomes clear that medial elongation does not depend on both tested elongasome components.

### Medial Elongation Requires FtsZ

**FIGURE 4:**
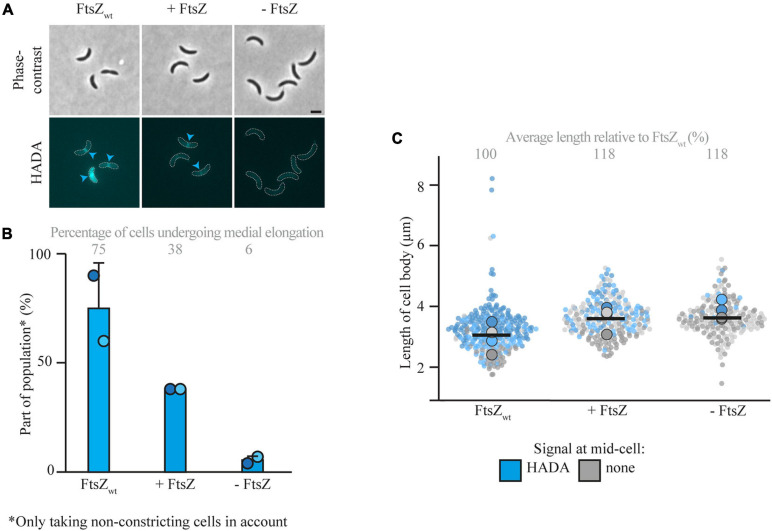
Medial elongation is affected by the absence of FtsZ. Light microscopy images **(A)** and respective quantification **(B)** of FtsZ depletion strain YB1585 (grown under depleting and inducing conditions) and MT309 (P_xyl_-*venus-mreB*), synchronized and subsequently grown for 45 min and subsequently subjected to a short pulse HADA staining. Cells in the representative images **(A)** are outlined and HADA midcell localization is indicated by blue arrowheads. Quantification of fluorescence signals **(B)** at midcell (using the color scheme outlined above) was performed on 100 non-constricting cells per replicate for two replicates per timepoint (value for each replicate is indicated by circles, replicate 1 is shown in a darker shade and replicate 2 in a light shade). Bars indicate the average of two replicates and error bars indicate standard deviation. In addition, the average percentage of non-constricting cells showing HADA is given for each timepoint. Data shown for FtsZ_wt_ in both cases are from the same replicates of strain MT309 as shown in Figure 1. Analysis of the cell length **(C)** of cells of strain YB1585 shows that they are slightly elongated as compared to cells harboring FtsZ_wt_. Cell lengths are shown in superplots, where each small dot indicates the value for a single cell. The color of each dot follows the color scheme outlined above, in order to identify differences in cell length for the different subpopulations, and shows different shades to differentiate between the replicates. The large dots show the average cell length for each localization subpopulation per replicate. The horizontal black bar indicates the average length over all cells and the numbers above the graph express this average length as a percentage of the average length of the cells carrying MreB_wt_ after 45 min (strain MT309, indicated in Figure 1).

After showing that medial elongation can take place in the absence of selected elongasome components, the focus was moved toward divisome components. Research in *E. coli* (de Pedro et al., 1997; Varma and Young, 2009; Potluri et al., 2012) and non-quantitative experiments in *C. crescentus* (Aaron et al., 2007) have implicated FtsZ in medial elongation. To investigate the requirement of FtsZ for medial elongation in more detail, cells of the FtsZ depletion strain YB1585 were cultivated and synchronized. After synchronization, cells were either grown in inducing (PYE with xylose) or depleting (PYE without xylose) conditions and PG incorporation was investigated *via* short pulse labeling with HADA 45 min after synchronization (Figure 4 and Supplementary Figure 4). As compared to a strain with wild-type amounts of FtsZ (MT309, grown in PYE without xylose), cells of YB1585 grown in both inducing and depleting conditions were slightly longer (Figure 4C) and were less likely to constrict (especially in the absence of FtsZ) (Supplementary Figure 4B), but showed less medial elongation (Figure 4B). The drop in fraction of cells undergoing medial elongation was especially clear for cells depleted of FtsZ, which contributed to only 6% of the population as opposed to 75% in cells with wild-type levels of FtsZ (MT309), demonstrating that FtsZ is required for medial elongation (Figures 4A,B).

## Discussion

The results obtained in this study suggest that medial elongation in *C. crescentus* requires FtsZ but not MreB at midcell, similar to PIPS in *E. coli* (de Pedro et al., 1997; Varma and Young, 2009; Potluri et al., 2012). Multiple experiments showed that medial elongation can take place upon MreB inhibition or its inability to get recruited to midcell. In this light, the finding that delayed recruitment of MreB to midcell results in a slower increase in the fraction of cells undergoing medial elongation is puzzling and needs further investigation. Possibly, this specific mutation in MreB has altered interaction properties with its binding partners, thereby affecting the process of medial elongation. The previous observation that the strain carrying this MreB variant has a longer cell cycle (Dye et al., 2011), might suggest all cells need to spend a certain time in the medial elongation phase before they can proceed to constriction.

The question which PG synthase(s) are involved in medial elongation in *C. crescentus* remains unanswered. The experiments presented here suggest PBP2 is not required and the same was suggested for PBP3 (Aaron et al., 2007), which again fits to what is described for PIPS in *E. coli*. PIPS in *E. coli* was reported to depend on two of its three bifunctional PBPs (bPBPs): PBP1A and PBP1B, that can take over each other’s role (Potluri et al., 2012; Pazos et al., 2018). *C. crescentus* has a higher redundancy of bPBPs with five copies in total: in addition to one PBP1C homolog (PbpZ), it interestingly has four PBP1A homologs (PBP1A, PbpC, PbpX, and PbpY), but lacks PBP1B homologs (Yakhnina and Gitai, 2013; Strobel et al., 2014). The observation that strains missing all bPBPs except for PbpX or PbpY or PbpC or to a lesser extent PPB1A are viable (Strobel et al., 2014), suggests that in *C. crescentus* PbpX, PbpY, PbpC, and potentially also Pbp1A might all be able to take up the role as bPBP involved in medial elongation.

Another open question when comparing medial elongation in *C. crescentus* to PIPS in *E. coli* is if any (and if so which) of the proteins anchoring FtsZ is involved in medial elongation. In *E. coli*, the early divisome protein ZipA works together with FtsA to anchor FtsZ to the membrane and stabilize FtsZ polymers into protofilaments (Pichoff and Lutkenhaus, 2002; Krupka et al., 2018). Both of these proteins are involved in PIPS, for which ZipA is essential unless a hyperactive FtsA allele is present (Potluri et al., 2012; Pazos et al., 2018). *C. crescentus*, however, lacks a ZipA homolog and FtsA is recruited only later to the divisome (Goley et al., 2011). Instead, the FtsZ-binding proteins ZapA, ZauP, FzlC, FtsE, and FzlA are recruited early to the divisome (Goley et al., 2011; Woldemeskel et al., 2017). If any of these proteins is required for medial elongation remains unknown at the moment. It might be that there is more redundancy in FtsZ anchoring proteins that are required for medial elongation compared to *E. coli*, as strains lacking ZapA, ZauP (and a combination of these two), FzlC, and FtsE are viable (Goley et al., 2010; Meier et al., 2017; Woldemeskel et al., 2017), suggesting that each of these alone does not take over the essential role of ZipA in medial elongation. Alternatively, the essential FzlA takes over the role of ZipA, although it is implicated in constriction and described to regulate the curvature of FtsZ filaments (Lariviere et al., 2018), which seems to occur at a later stage than the role ZipA takes in PIPS. All in all, further studies need to be performed in order to understand which other proteins are involved in medial elongation. Until these proteins and the underlying mechanisms they use to make *C. crescentus* cells elongate from midcell are known, it will be impossible to answer if this growth phase is mechanistically the same as PIPS in *E. coli*. For now, however, all results seem consistent. Hopefully research in the future will succeed in elucidating mechanisms behind this elusive growth phase and answer the question how widespread it is in bacteria.

## Data Availability Statement

The raw data supporting the conclusions of this article will be made available by the authors, without undue reservation.

## Author Contributions

MvT conceived and designed this project, performed the analyses, and wrote the manuscript.

## Conflict of Interest

The author declares that the research was conducted in the absence of any commercial or financial relationships that could be construed as a potential conflict of interest.

## Publisher’s Note

All claims expressed in this article are solely those of the authors and do not necessarily represent those of their affiliated organizations, or those of the publisher, the editors and the reviewers. Any product that may be evaluated in this article, or claim that may be made by its manufacturer, is not guaranteed or endorsed by the publisher.
