# Supplementary material for: Elongation at Midcell in Preparation of Cell Division Requires FtsZ, but Not MreB nor PBP2 in Caulobacter crescentus
Source: Front Microbiol. 2021 Aug 27;12:732031. doi: 10.3389/fmicb.2021.732031 (PMC8429850; doi:10.3389/fmicb.2021.732031)
Supplement: Supplementary file 1 [file Data_Sheet_1.DOCX]

Supplementary Material for:

Elongation at midcell in preparation of cell division requires FtsZ, but not MreB nor PBP2 in *Caulobacter crescentus*

Muriel C.F. van Teeseling^1,2*^


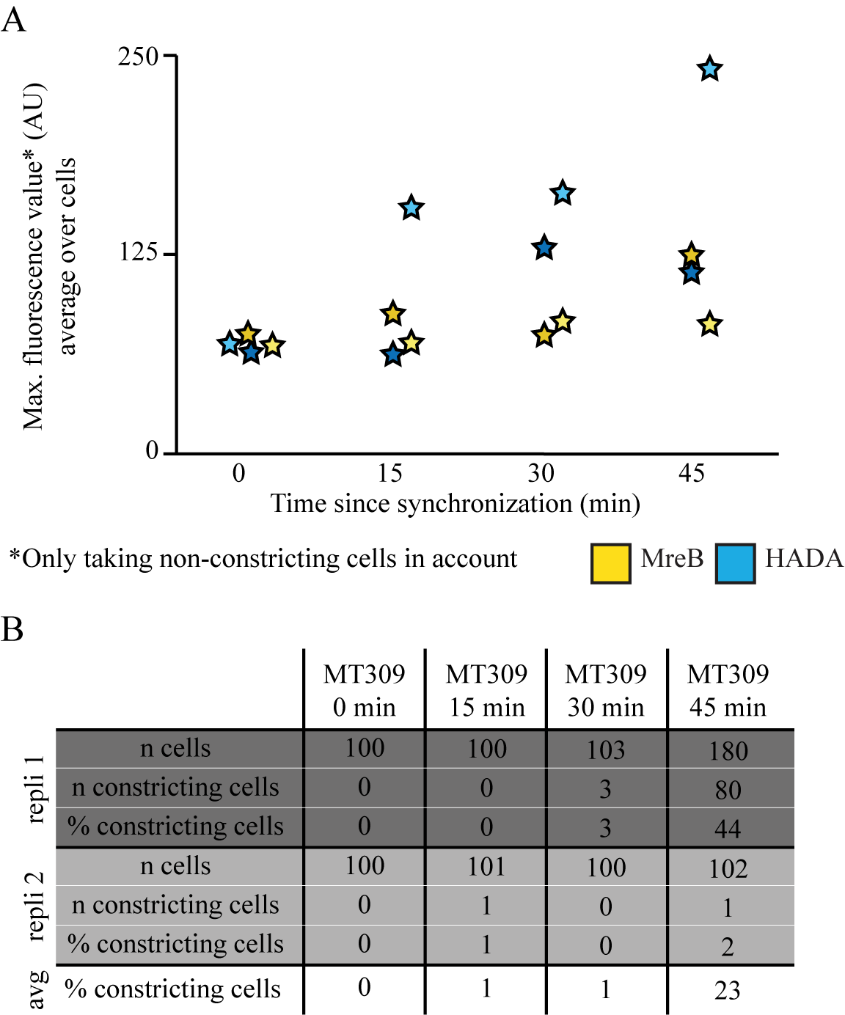


**Supplementary Figure 1.** Local peptidoglycan incorporation (maximum HADA signal intensity) becomes more prominent over time. Quantification (A) of maximum intracellular fluorescence intensities of Venus(-MreB) (yellow stars) and HADA (blue stars) averaged over 100 cells per replicate (replicate 1 indicated by a darker shaded star and replicate 2 by a lighter shaded star) of strain MT309 (P_xyl_-*venus-mreB*) grown for the indicated duration after synchronization, stained with a short pulse of HADA. Number of cells showing constriction increases with time (B). Shown are the amount of cells analysed per replicate to get to 100 non-constricting cells for each replicate. Also indicated are the number of constricting cells per replicate, as well as the percentage of cells undergoing constriction both per replicate and the average of the two replicates. The total number of cells analysed per replicate (n cells) reflects the number of cells shown in the superplots in Fig.1.

**
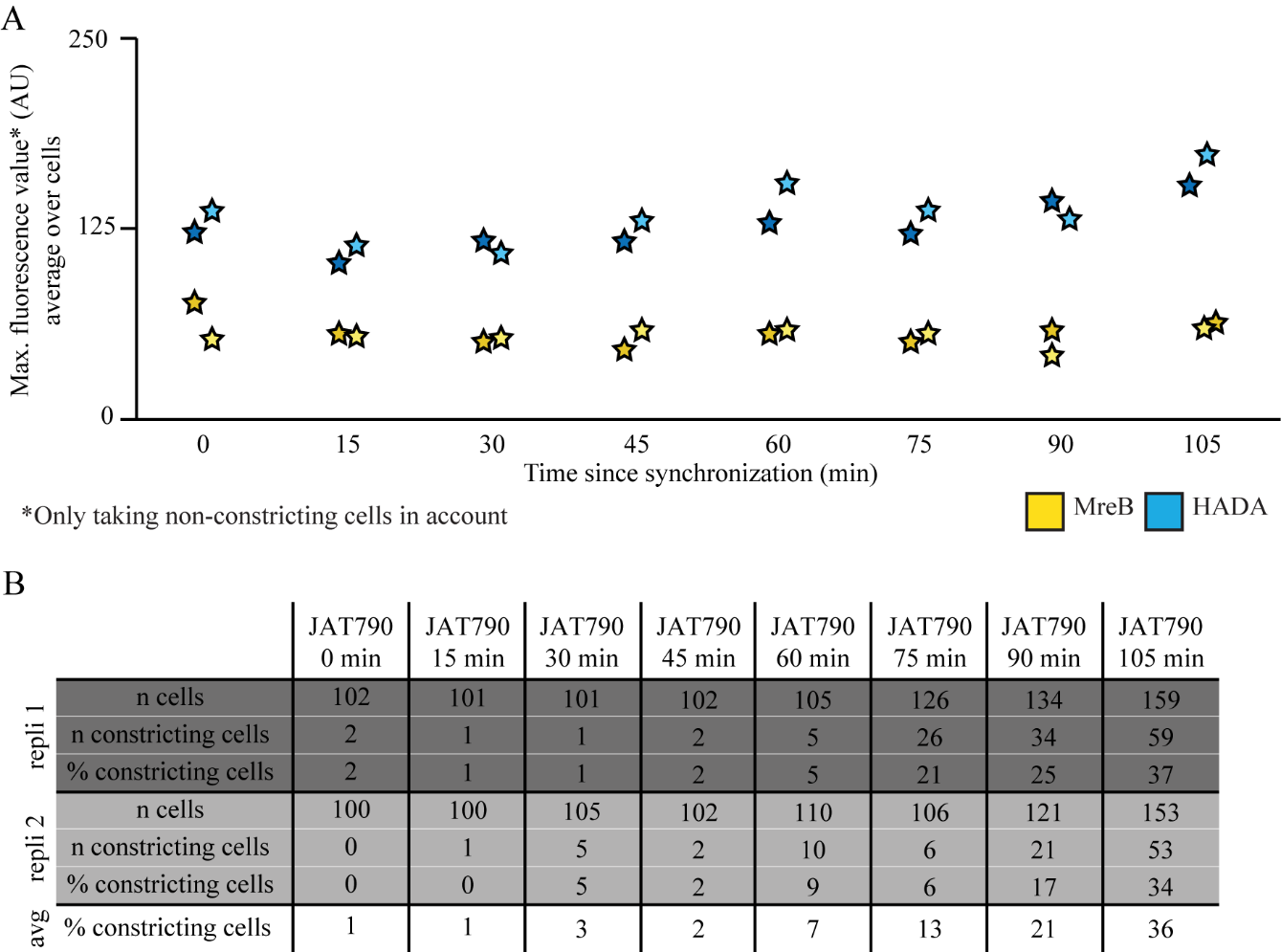
**

**Supplementary Figure 2.** Local peptidoglycan incorporation (maximum HADA signal intensity) becomes slightly more prominent over time. Quantification (A) of maximum intracellular fluorescence intensities of _G165A_Venus(-MreB) (yellow stars) and HADA (blue stars) averaged over 100 cells per replicate (replicate 1 indicated by a darker shaded star and replicate 2 by a lighter shaded star) of strain JAT790 (P_mreB_-*_G165A_mreB* P_xyl_-*venus-_G165A_mreB*) grown for the indicated duration after synchronization, stained with a short pulse of HADA. Number of cells showing constriction increases with time (B) considerably slower than in strain MT309 (Suppl. Fig. 1). Shown are the number of cells analysed per replicate to get to 100 non-constricting cells for each replicate. Also indicated are the number of constricting cells per replicate, as well as the percentage of cells undergoing constriction both per replicate and the average of the two replicates. The total number of cells analysed per replicate (n cells) reflects the number of cells shown in the superplots in Fig.2.


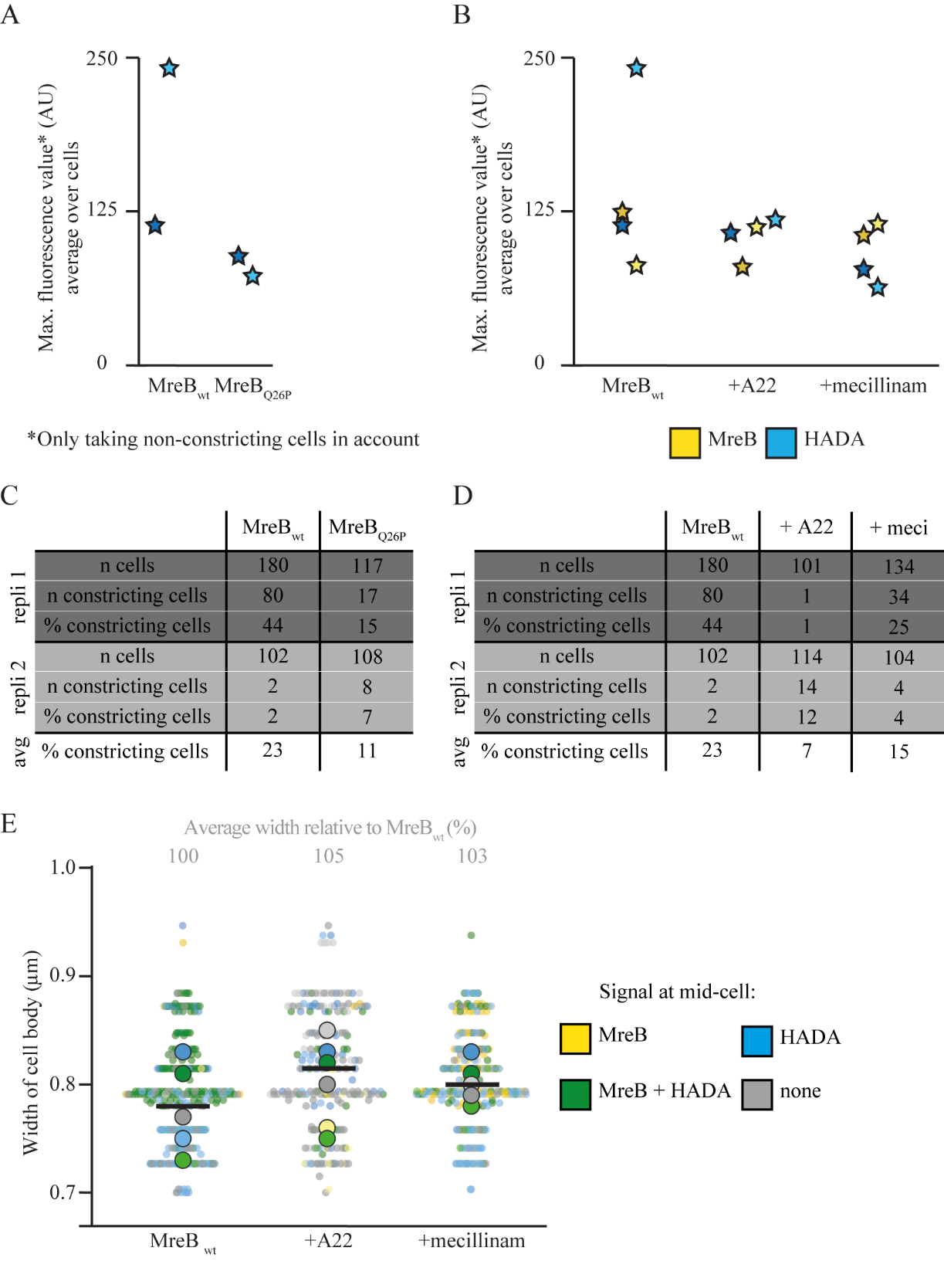


**Supplementary Figure 3.** Local peptidoglycan incorporation (maximum HADA signal intensity) under different conditions. Quantification (A&B) of maximum intracellular fluorescence intensities of Venus(-MreB) (yellow stars) and HADA (blue stars) averaged over 100 cells per replicate (replicate 1 indicated by a darker shaded star and replicate 2 by a lighter shaded star) of strains CJW1715 (P_mreB_- *_Q26P_mreB*) (A) and MT309 (P_xyl_-*venus-mreB*), synchronized and subsequently grown in the absence of additives and (for MT309) in the presence of the MreB inhibitor A22 (10 µg/ml) or the PBP2 inhibitor mecillinam (150 µg/ml) (B). Cells were harvested 45 min after synchronization and subjected to a short pulse HADA staining. Number of cells showing constriction under different conditions (C&D). Shown are the number of cells analysed per replicate to get to 100 non-constricting cells for each replicate. Also indicated are the number of constricting cells per replicate, as well as the percentage of cells undergoing constriction both per replicate and the average of the two replicates. The total number of cells analysed per replicate (n cells) reflects the number of cells shown in the superplots in Fig.3 and panel (E) of this figure. Cell width increases upon treatment with drugs inhibiting MreB and PBP2 (E). Cell widths are shown in superplots, where each small dot indicates the value for a single cell. The color of each dot indicates the if MreB and HADA signal are seen at midcell (green), only HADA (blue) or only MreB (yellow), in order to identify differences in cell width for the different subpopulations, and shows different shades to differentiate between the replicates. The large dots show the average cell width for each localization subpopulation per replicate. The horizontal black bar indicates the average width over all cells and the numbers above the graph express this average width as a percentage of the average width of the cells carrying MreB_wt_ after 45 min (strain MT309). Number of cells included in the superplots are indicated in panel D. Data shown for MreB_wt_ in both cases are from the same two replicates of strain MT309 (also shown in Suppl. Fig. 1).


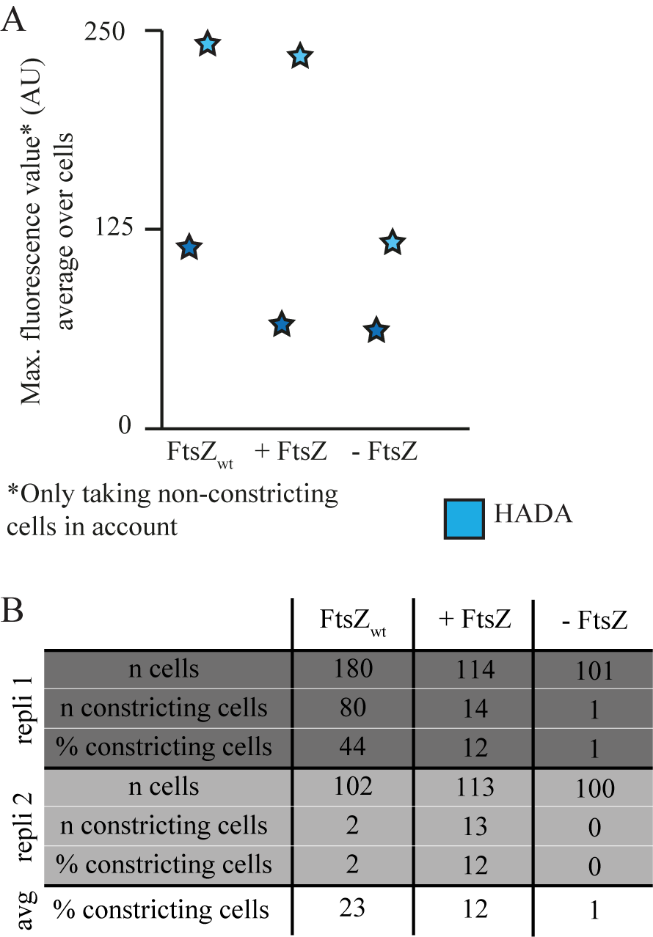


**Supplementary Figure 4.** Local peptidoglycan incorporation (maximum HADA signal intensity) decreases in the absence of FtsZ. Quantification (A) of maximum intracellular fluorescence intensities of HADA (blue stars) averaged over 100 cells per replicate (replicate 1 indicated by a darker shaded star and replicate 2 by a lighter shaded star) of FtsZ depletion strain YB1585 (grown under depleting and inducing conditions) and strain MT309 (P_xyl_-*venus-mreB*) grown for 45 min and subsequently subjected to a short pulse HADA staining. Number of cells showing constriction is lower in the absence of FtsZ (B). Shown are the number of cells analysed per replicate to get to 100 non-constricting cells for each replicate. Also indicated are the number of constricting cells per replicate, as well as the percentage of cells undergoing constriction both per replicate and the average of the two replicates. The total number of cells analysed per replicate (n cells) reflects the number of cells shown in the superplots in Fig.4. Data shown for FtsZ_wt_ in both cases are from the same two replicates of strain MT309 (also shown in Suppl. Fig. 1).
